# Test-Retest Reliability of Web-Based Retrospective Self-Report of Tobacco Exposure and Risk

**DOI:** 10.2196/jmir.1248

**Published:** 2009-08-11

**Authors:** Janet Brigham, Christina N Lessov-Schlaggar, Harold S Javitz, Ruth E Krasnow, Mary McElroy, Gary E Swan

**Affiliations:** ^2^Washington University School of MedicineSt. LouisMOUSA; ^1^Center for Health SciencesSRI InternationalMenlo ParkCAUSA

**Keywords:** Tobacco smokers, retrospective studies, psychometrics

## Abstract

**Background:**

Retrospectively collected data about the development and maintenance of behaviors that impact health are a valuable source of information. Establishing the reliability of retrospective measures is a necessary step in determining the utility of that methodology and in studying behaviors in the context of risk and protective factors.

**Objective:**

The goal of this study was to examine the reliability of self-report of a specific health-affecting behavior, tobacco use, and its associated risk and protective factors as examined with a Web-based questionnaire.

**Methods:**

Core tobacco use and risk behavior questions in the Lifetime Tobacco Use Questionnaire—a closed, invitation-only, password-controlled, Web-based instrument—were administered at a 2-month test-retest interval to a convenience sample of 1229 respondents aged 18 to 78 years. Tobacco use items, which covered cigarettes, cigars, smokeless tobacco, and pipe tobacco, included frequency of use, amount used, first use, and a pack-years calculation. Risk-related questions included family history of tobacco use, secondhand smoke exposure, alcohol use, and religiosity.

**Results:**

Analyses of test-retest reliability indicated modest (.30 to .49), moderate (.50 to .69), or high (.70 to 1.00) reliability across nearly all questions, with minimal reliability differences in analyses by sex, age, and income grouping. Most measures of tobacco use history showed moderate to high reliability, particularly for age of first use, age of first weekly and first daily smoking, and age at first or only quit attempt. Some measures of family tobacco use history, secondhand smoke exposure, alcohol use, and religiosity also had high test-retest reliability. Reliability was modest for subjective response to first use.

**Conclusions:**

The findings reflect the stability of retrospective recall of tobacco use and risk factor self-report responses in a Web-questionnaire context. Questions that are designed and tested with psychometric scrutiny can yield reliable results in a Web setting.

## Introduction

### Studying Behavior in Context

Behaviors that can impact health are not isolated phenomena, separate from other behaviors and independent of forces influencing decisions and outcomes. Consequently, the use of tobacco and other potentially harmful substances is often studied in relation to the lifetime context of use. For example, the Rasch model analysis of smoking and alcohol use [1] reflects the intertwined relationships among different substances, as well as the advantage of studying multiple substances and risk factors in concert. Recent research focusing on aspects of social network that affect lifetime tobacco cessation outcomes [2] underscores the desirability of examining substance use in larger social and cultural contexts.

The contextual setting of tobacco use involves risk or protective factors that can affect tobacco use. These factors, as summarized by Sussman [3], can be examined readily through self-report and retrospective questioning. Factors include education, income, race and ethnicity, family use and peer use of tobacco, perceived consequences, access to tobacco, opportunities for use, cognition, habits, and addictions.

### Retrospective Research

Relevant information about tobacco use is often not collected at the time events occur. This necessitates retrospective research, which has been scrutinized as a means of collecting information relating to lifetime patterns of tobacco use. Retrospective data collection allows exploration of events that may not have been perceived as important at the time they occurred. For example, a contemporary researcher desiring to study changes in tobacco initiation ages across several decades probably would need to use retrospective techniques.

However, not all questions are amenable to retrospective inquiry, as Kenkel and colleagues [4] indicated. In examining the usefulness of retrospective measures of smoking from national survey samples, they reported that specificity in questions and statistical methods was critical for obtaining accurate retrospective measurements; they concluded that some aspects of tobacco use, such as frequent, temporary quit attempts, were not amenable to retrospective study, although the data could be helpful for studying more prolonged or permanent events. Johnson and Mott [5], studying age of onset of tobacco, alcohol, and other substance use, concluded that retrospective information typically obtained through questionnaires was adequate for most epidemiological applications. Reliability of reported age at onset [6] can benefit from aided recall and contextual information.

### Reliability

Retrospective and contemporaneous examinations of risk and protective factors have demonstrated mixed psychometric adequacy. Post and colleagues [7] reported that maternal retrospective recall of smoking during pregnancy was “fairly stable over time,” concurring with an earlier study by Matt and colleagues [8]. Grant and coauthors [9] studied reliability of numerous substance use disorders and associated behaviors, finding moderate to high reliability for tobacco use measures in a test-retest interval of 2 to 10 weeks. Reliability of reporting of family history of depression was high regarding parents’ and siblings’ conditions. Ruan and colleagues [10] found moderate to high reliability for addiction risk factor measures in 2- to 10-week test-retest. Reliability was highest for recent stressful events and stigma of alcoholism.

Self-described religiosity has been identified as a protective factor capable of attenuating an additive genetic risk for smoking initiation and thus moderating genetic influences on the liability for smoking [11]. A study of measures of spirituality, mindfulness, and substance use [12] reported moderate to high reliability for assessment of religiosity and spirituality.

These varied reports provide support for the feasibility of examining the psychometric properties of risk and protective factors related to substance use. These findings also reflect the potential utility of assessing tobacco use in the context of life events. The present study examined the reliability of retrospective questions about tobacco exposure and factors that could influence tobacco use. The instrument was a Web-based questionnaire designed to minimize error and maximize respondent involvement.

### Research Goals

A primary goal of the present study was to examine 2-month test-retest reliability of retrospective self-report of tobacco use and risk-related behaviors. A previous study by the present authors [13] identified moderate to high 2-year test-retest reliability of items about lifetime tobacco use. In view of standards for reliability testing [14-16] and the positive findings from that longer-interval study, the present study addressed the following research questions: (1) How reliable is recall of tobacco use and elements of risk and protection? and (2) What factors moderate the reliability of recall?

A related goal was to continue to explore questionnaire reliability of a Web self-administration instrument. As Internet access expands [17], questions arise about the representativeness and generalizability of Web samples. We explored the reliability of Web administration within the framework of a closed, invitation-only, passcode-controlled Web-based questionnaire.

## Methods

### Recruitment

The Institutional Review Board of SRI International of Menlo Park, California, approved the study and determined that it was exempt from requirements for informed consent because respondents’ identity was anonymous to the researchers and the responses presented no risk of jeopardy. Signed informed consent was not required. As described in the following paragraphs, participants were invited to participate in a study about tobacco use and were provided contact information for the investigators and for technical support. The study was identified on the introductory screen and succeeding screens as being sponsored by SRI International.

The sample size goal of 1200 respondents was established based on consideration of the half-width of confidence intervals for relevant statistics. This sample size was sufficient so that (1) the 95% confidence interval for a percent responding in a category at a given time would have a half-width no greater than 0.03, (2) the confidence interval for a Pearson correlation statistic of .50 for normally distributed variables would have a half-width no greater than .04, and (3) for a dichotomous variable with 75% agreement (evenly divided between agreement on each value of the variable) and a kappa statistic of 0.50, the 95% confidence interval for the true kappa would be no greater than 0.05.

The Web-based questionnaire, the Lifetime Tobacco Use Questionnaire (LTUQ), was self-administered two times, 2 months apart, by a randomly selected, invitation-only convenience sample of adults aged 18 and older drawn from a US consumer panel (e-Rewards Inc., Dallas, TX, USA). The Web panel comprised millions of persons invited from consumer databases, such as public utility customers; airline, dining, and hotel program members; and other customer groups. Opt-in membership was not allowed either to the panel or to the study.

The data were collected in two waves because of budget allocations. Nearly identical waves of 2-month test-retest administration occurred in January/March 2006, and August/October 2006. The January and August 2006 administrations were referred to as Time 1 and were grouped for statistical analysis. The March and October 2006 administrations were grouped and referred to as Time 2.

Time 1 invitations were emailed to randomly selected members of the consumer panel. Reminder invitations were sent 1 week later to those invitees who had not yet completed the LTUQ. For retesting at Time 2, all Time 1 respondents were invited to re-take the LTUQ. Time 1 respondents not responding to the first Time 2 invitation within 1 week were sent a second invitation. Test-retest duration approximated 2 months, with variation ± 2 weeks.

### Administration

Respondents’ identity remained anonymous to the investigators. Respondents self-administered the questionnaire through a passcode-controlled website and could suspend the questionnaire and resume at their convenience using a passcode. The incentive was US$10 in e-Rewards scrip, the standard mechanism of payment to e-Rewards panel members.

Each respondent received a unique passcode to self-administer the questionnaire through a secure website. Cookies were not used, and IP addresses were not available to the investigators. SRI International researchers received all data without personal identifiers. Only the Web sample provider knew the respondents’ identities. The sample provider did not have access to the LTUQ data and could not connect the respondents’ identities with their responses. Data were encoded and collected on secure central servers and later decoded by the software provider (WebSurvent, CfMC, San Francisco, CA, USA) before the data were provided to the investigators.

### Measures

The LTUQ [13] retrospectively assessed the use of any form of tobacco or nicotine across the lifespan. Developed initially in 1998, the LTUQ was tested in three earlier versions on more than 4000 respondents through computer-assisted self-interviewing (CASI), computer-assisted telephone interviewing (CATI), and computer-assisted personal interviewing (CAPI), and usability testing was conducted prior to the present CASI study. The programming utilized computerized features including skip logic, branching, and loops to shorten testing time and minimize attrition. Response options were randomized and rotated to reduce sequence effects and carryover/practice effects, with some response options anchored for consistency. The questionnaire included internal validity checks, accuracy checks, and response limitations that either prevented respondents from entering certain types of inaccurate data or flagged those responses for later examination. Because of these features, the LTUQ cannot be administered in noncomputer mode (see Multimedia Appendix 1).

A progress bar indicated the approximate percent completion of the survey as respondents proceeded through the questions. Respondents could review all prior questions and could change their responses prior to completion. Only completed questionnaires were used in the data analyses.

The LTUQ was structured around a core questionnaire that assessed the extent and nature of tobacco use from earliest exposure to the point of testing. Questions covered four major types of tobacco—cigarettes, cigars, smokeless tobacco, and pipe tobacco—and included an open-ended response option for other tobacco-delivery methods such as waterpipe or bidi. In addition to the core questions, module questions examined risk and protective factors related to tobacco use.

The core tobacco-use questions assessed initial use, transition to regular daily or weekly use, regular use, dependence, quit attempts, and abstinence. Modules of additional questions addressed (1) subjective reactions to initial use, (2) secondhand smoke exposure, (3) familial use of tobacco, (4) alcohol use, and (5) religiosity. Pack-years of smoking cigarettes was calculated from questions about the extent and duration of cigarette use and periods of abstinence.

Several minor typographical and programming errors were corrected for the August/October testing, and several additional risk-related questions were appended near the end of the questionnaire for the August/October testing.

#### Respondent Characteristics

Demographic characteristics of age, sex, and an estimate of median household income were evaluated as independent variables potentially affecting reliability. Since age and sex were screening variables for identifying invalid test-retest responses, reliability estimates were not calculated for those variables. Race/ethnicity and education data also were obtained but were not assessed for effects on reliability.

Respondents were grouped into terciles for examination of age effects on the reliability of recall. Age groupings were determined through the SAS procedure PROC RANK, to establish three groups of approximately the same size. The three similarly sized age groups were as follows: younger (18 to 37 years old, n = 422, mean = 30.8 years, SD = 4.2), middle (38 to 50 years old, n = 400, mean = 44.2 years, SD = 3.8), and older (51 to 78 years old, n = 402, mean = 57.6 years, SD = 5.4). Item reliabilities were calculated within each age group and compared using the chi-square test.

Median household income (in US dollars) was estimated from 2000 US Census ZIP codes [18], separated by terciles: lower ($16,383 to $41,430, n = 398, mean = $34,530, SD = $5160), middle ($41,554 to $56,585, n = 398, mean = $48,678, SD = $4466), and higher ($56,589 to $140,357, n = 396, mean = $70,895, SD = $13,200).

#### Tobacco Use

Questions about overall tobacco use included smoking 100 cigarettes in lifetime, frequency of use of all tobacco types, and current use.

Measures related to the first use of tobacco included (1) age at first tobacco use, (2) type of tobacco first used, (3) amount used at first exposure, and (4) subjective reactions to first tobacco use (dizzy, lightheaded, nauseated, enjoyed it, coughing/choking, liked taste, felt bad, relaxed/calm, irritated throat or lungs, head rush or buzz, felt good, difficulty inhaling, and liked smell) rated on a scale from 1 (“not at all”) to 5 (“very much”) or “unsure.”

Lifetime frequency of tobacco use was assessed for each tobacco type (cigarettes, cigars, smokeless tobacco, pipe tobacco, other) on a 5-point scale ranging from “never used” to “used at least daily for at least 1 month.” Additionally, when respondents indicated at least weekly or daily use, the frequency and amount of daily and weekly tobacco use were assessed with questions regarding (1) age at onset of weekly/daily tobacco use and (2) amount of tobacco used weekly/daily after the onset of weekly/daily use.

Current tobacco use was assessed for four primary types of tobacco (cigarettes, cigars, smokeless tobacco, pipe tobacco) plus other types. Dependence was assessed for onset of daily use of cigarettes. Quitting history included first and most recent quit attempt of at least 3 months’ duration, allowing for brief lapses.

Pack-years typically is calculated by multiplying the number of packs of cigarettes smoked per day by the number of years an individual has smoked [19]. We did not calculate a comparable measure for tobacco types other than cigarettes [20] because of low use (see Table 1). We calculated pack-years in detail by averaging amount smoked across periods of known use, excluding periods of abstinence of at least 3 months’ duration. Pack-years calculation was possible for only part of the subject sample because questions facilitating its calculation were added for the August/October respondents.

#### Risk and Protective Factors for Tobacco Use

Questions on family history of tobacco use were based on a family smoking index [21] that asked about paternal, maternal, sibling, and offspring use of tobacco.

Regarding secondhand smoke exposure, respondents were questioned about current home and vehicle rules and about children’s exposure to secondhand smoke.

Alcohol use was probed with questions about ever use, age at first use, use of alcohol and tobacco together, and extent of alcohol use.

Religiosity questions were based on the Intrinsic Religious Motivation Scale [22-24], with additional questions regarding attendance at religious meetings, prayer/meditation, and participation in groups discouraging tobacco use.

### Data Analyses

Analyses were calculated using SAS (SAS Institute Inc., Cary, NC, USA). Frequencies, means, percentages, standard deviations, and correlations were conducted as standard descriptive statistics. Test-retest reliability for dichotomous and categorical items was computed using the kappa statistic (k) for categorical data [25]; for ordinal and continuous measures, test-retest reliability was computed using the intraclass correlation coefficient (ICC). Reliability was rated as modest (.30 to .49), moderate (.50 to .69), or high (.70 to 1.00) for the purposes of comparison. Some demographic differences were examined with the chi-square test. Differences in test-retest reliability in men and women were compared using a 2-tailed *t* test of equality of means applied to point estimates of reliability and their asymptotic variance estimates.

We did not employ weighting techniques to match the US population or the US tobacco-user population since we were not attempting to describe population characteristics with this convenience sample.

The responses “don’t know” and “unsure” were included in some analyses (indicated in table footnotes) where those responses were potentially informative about difficulty of recall, such as a “don’t know” response to a question about age of first alcohol use.

## Results

Median time to completion of the questionnaire was 13.7 minutes at both Time 1 and 2. Median was a more useful measure than mean because of the likelihood that respondents left the questionnaire while engaging in other activities.

### Data Integrity

Responses to scaled grid questions were evaluated for the presence of straight-line responding other than “unsure/don’t know” options and examined for excessively short response times. A total of 24 out of 1253 respondents at both Time 1 and Time 2 were excluded for multiple mismatches and other indices of inadequate responding [26]. Five data-point outliers excluded in data analyses (indicated in table footnotes) ranged from 40 to 2582 standard deviations from the mean and appeared to be inaccurate responses to single questions rather than intentionally incorrect responses as part of a pattern of inadequate responding (see Multimedia Appendix 2).

### Response Rate

Respondents at Time 1 (N = 3142) were re-invited at Time 2; those responding to the Time 2 retest invitation and completing the LTUQ (N = 1229, 39.1% response rate, see [27]) were included in the analyses. Nonresponse due to changes in email address, Internet access, or other factors could not be determined.

Differences between Time 2 responders and nonresponders were examined on several dimensions of demographics and tobacco use. Time 1 respondents not responding at Time 2 were more likely to be slightly younger (mean = 42.7 years, SD = 12.2 for nonresponders vs mean = 44.0 years, SD = 11.9 for responders; *t* = −2.93, *P* = .003), more likely to be female (55.1%, 1068 of 1914 nonresponders vs 51.6%, 632 of 1229 responders; χ^2^ = 9.6, *P* = .008), less likely to report race as white (85.8%, 1642 of 1914 nonresponders vs 87.6%, 1077 of 1229 responders; χ^2^ = 6.5, *P* = .04), and more likely to have smoked at least 100 cigarettes in their lifetime (98.9%, 1888 of 1910 nonresponders vs 96.0%, 1175 of 1224 responders; χ^2^ = 27.4, *P* < .001).

### Test-Retest Reliability Estimates

Most reliability estimates calculated on the test-retest sample were statistically significant, although reliability was modest for some measures.

#### Respondent Characteristics

Respondents included in the test-retest analyses (Time 2, N = 1229) ranged in age at Time 1 from 18 to 78 years (Table 1). Less than 1% (5 of 1229) reported never using tobacco or nicotine; their data were included if questions did not require exposure to self-administered tobacco or nicotine. About 84% (926 of 1102) of respondents reported having used cigarettes either daily or weekly, with minimal use of cigars, smokeless tobacco, or pipe tobacco. Subjects self-reported demographic information regarding education and race/ethnicity with high reliability (Table 1).

#### Tobacco Use

Some measures relating to lifetime tobacco use and specifically to cigarette use showed high reliability (Table 1 and Table 2). This included smoking more than 99 cigarettes in lifetime, current cigarette use, age at first use, age at first weekly and daily use, age at and duration of first or only quit attempt, and lifetime pack-years.

Although reliability of test-retest self-report of the age of first tobacco use (mean = 15.5 years reported at Time 1 and Time 2) was high, other aspects of first use reflected modest to moderate reliability. Test-retest reliability was moderate for type of tobacco first used, which reportedly was a cigarette for about 94% (1092 of 1162) of participants. Subjective responses to first use had modest to moderate reliability (Table 2).

Separate sets of questions asked about the age of first weekly smoking and the amount used at that time, and the age of first daily smoking and the amount used at that time. Reliability was higher for age at onset of weekly or daily use than for the number of cigarettes used, which had moderate reliability. Dependence-related questions regarding the time to first cigarette in the morning at the onset of daily cigarette use had moderate reliability (Table 2). Age at first or only quit attempt of at least 3 months’ duration exhibited high reliability, as did the duration of that quit attempt and the use of a cessation aid (Table 2).

Test-retest calculation of pack-years, a common metric for evaluating tobacco use across the lifespan, was evaluated in the August/October group only because a question added mid-study made the pack-years calculations possible. Reliability of the pack-years calculation was high (Table 2).

**Table 1:** Test-retest reliability of respondents’ self-report of demographics and tobacco use

		Time 1	Time 2	No.	ICC or κ^a^	95% CI or SE
**Demographics**						
	Age, years, mean (SD)	44.0 (11.9)	44.1 (11.9)	1229		
	Female, %	51.6	51.5	1229		
	Education: > high school, %	88.9	89.0	1229	κ = 0.88	0.86, 0.90
	Ethnicity: white, %	87.6	87.9	1229	κ = 0.86	0.82, 0.90
**Lifetime use of cigarettes**						
	Smokers reporting using > 99 cigarettes/lifetime, %	96.22	96.14	1217	κ = 0.81	0.72, 0.90
	Total cigarettes smoked if < 100/lifetime, mean (SD)	19.3 (25.1)	22 (23.7)	38	ICC = 0.70	0.08
**Frequency of tobacco use**						
	Cigarettes daily, % (no.)	83.7 (922)	84.0 (926)	1102	κ = 0.51	0.44, 0.57
	Cigars, % ever weekly or daily (no.)	6.6 (73)	5.5 (61)	1102	κ = 0.66	0.63, 0.70
	Smokeless tobacco, % ever weekly or daily (no.)	2.8 (31)	2.7 (30)	1102	κ = 0.71	0.66, 0.76
	Pipe tobacco, % ever weekly or daily (no.)	2.5 (28)	2.0 (22)	1102	κ = 0.70	0.66, 0.74
	Other tobacco/nicotine, % (no.)	6.2 (68)	5.3 (58)	1102	κ = 0.50	0.38, 0.61
**Current tobacco use**						
	Number of cigarettes/week, mean (SD)	103.7 (88.1)	103.7 (89.4)	859	ICC = 0.83	0.01
	Number of cigars/week, mean (SD)	0.91 (8.8)	1.19 (9.9)	858	ICC = 0.30^b^	0.03
	Number of tins of smokeless tobacco/week^b^, mean (SD)	0.07 (0.6)	0.09 (0.7)	858	ICC = 0.90	0.01
	Number of pipe tobacco uses/week, mean (SD)	0.14 (2.2)	0.15 (2.2)	858	ICC = 0.98^b^	0.001

^a^ Reliability was not calculated for age and sex because those variables were used for screening.

^b^ ICC calculations excluded one outlier.

**Table 2:** Test-retest reliability of self-report details of tobacco use history

		Time 1	Time 2	No.	ICC or κ	95% CI or SE
**First useof tobacco**						
	Age first tried tobacco, years, mean (SD)	15.5 (3.5)	15.5 (3.9)	1162	ICC = 0.81	0.01
	First tobacco was cigarette, %	94.2	93.4	1162	κ = 0.51	0.38, 0.64
**Experience at first use of tobacco (1–5 scale), mean (SD)**						
	Dizzy	3.14 (1.3)	3.19 (1.3)	1162	ICC = 0.51	0.02
	Lightheaded	3.10 (1.3)	3.15 (1.3)	1162	ICC = 0.49	0.02
	Nauseated	2.21 (1.3)	2.28 (1.3)	1162	ICC = 0.54	0.02
	Enjoyed it	3.00 (1.1)	2.87 (1.1)	1162	ICC = 0.50	0.02
	Coughing/choking	2.91 (1.3)	2.97 (1.3)	1162	ICC = 0.51	0.02
	Liked taste	2.56 (1.2)	2.51 (1.1)	1162	ICC = 0.51	0.02
	Felt bad	2.19 (1.2)	2.24 (1.2)	1162	ICC = 0.51	0.02
	Relaxed/calm	2.70 (1.2)	2.61 (1.1)	1162	ICC = 0.36	0.03
	Irritated throat	2.64 (1.3)	2.73 (1.3)	1162	ICC = 0.49	0.02
	Head rush/buzz	3.36 (1.3)	3.47 (1.2)	1162	ICC = 0.52	0.02
	Felt good	2.58 (1.2)	2.51 (1.1)	1162	ICC = 0.38	0.03
	Difficulty inhaling	2.59 (1.4)	2.69 (1.4)	1162	ICC = 0.41	0.03
	Liked smell	2.44 (1.2)	2.46 (1.2)	1162	ICC = 0.51	0.02
**Weekly useof cigarettes**						
	Age first smoked cigarettes at least weekly, years, mean (SD)	17.3 (4.2)	17.3 (4.1)	913	ICC = 0.85	0.01
	Number of cigarettes/week when started weekly use, mean (SD)	34.3 (35.1)	34.2 (39.3)	554	ICC = 0.52	0.03
**Daily use of cigarettes**						
	Age first used daily, years, mean (SD)	17.6 (4.5)	17.6 (4.3)	857	ICC = 0.82	0.01
	Number of cigarettes/day when started daily use, mean (SD)	9.64 (7.2)	9.50 (7.3)	515	ICC = 0.54	0.03
	Smoked < 1 hour after waking when started daily use, %	26.3	26.5	515	κ = 0.53	0.44, 0.62
	Minutes to first cigarette of day when started daily use, mean (SD)	160.4 (148)	148.6 (137)	411	ICC = 0.57	0.03
**First or only cigarette quit attempt of** ≥ **3 months’ duration**						
	Age, years, mean (SD)	28.3 (9.7)	28.2 (9.8)	546	ICC = 0.86	0.01
	Number of months, mean (SD)	13.5 (17.6)	12.9 (16.6)	430	ICC = 0.79	0.02
	Used cessation aid, %	25.1	24.0	546	κ = 0.71	0.64, 0.78
**Pack-years**						
	Pack-years, mean (SD)	19.1 (18.1)	19.9 (18.7)	504	ICC = 0.76	0.02

#### Risk and Protective Factors for Tobacco Use

Reliability was moderate or high for questions about the four risk/protective categories: family history of tobacco use, secondhand smoke exposure, alcohol use, and religiosity (Table 3). Reliability of family history reports of parental, sibling, and offspring tobacco use were high. Questions about exposure to secondhand smoke indicated moderate to high reliability. Questions about alcohol use ranged in reliability from moderate to high. Respondents indicated at both Time 1 and Time 2 that when they drank alcohol, they also used tobacco about 60% of the time. Among questions about religiosity, those indicating highest reliability were regarding seeking divine guidance in decision making, and serving God (Table 3). Reliability of other questions regarding religiosity ranged from modest to high.

**Table 3:** Test-retest reliability of measures of risk for and protection against tobacco use^a^

		Time 1	Time 2	No.	ICC or κ	95% CI or SE
**Family historyof tobacco use**						
	Mother used tobacco, %	47.8	48.6	1188	κ = 0.86	0.83, 0.89
	Father used tobacco, %	72.4	74.0	1148	κ = 0.75	0.71, 0.79
	Number of siblings who used tobacco, mean (SD)	1.38 (1.5)	1.40 (1.5)	392	ICC = 0.84	0.02
	Number of offspring who used tobacco, mean (SD)	0.68 (1.0)	0.63 (0.9)	243	ICC = 0.87	0.02
**Exposure to secondhand smoke**						
	Smoking currently allowed inside home, % no	56.0	57.2	1213	κ = 0.80	0.77, 0.83
	Smoking currently allowed in car, % no	38.2	38.4	1213	κ = 0.83	0.80, 0.86
	Children currently exposed to smoke inside home, % no	85.6	87.0	1229	κ = 0.65	0.58, 0.71
**Alcohol use**						
	Ever used alcohol, %	91.2	89.3	522	κ = 0.41	0.29, 0.54
	Drink alcohol currently, %	80.4	75.9	515	κ = 0.59	0.50, 0.67
	Age first tried alcohol, years, mean (SD)	15.9 (2.8)	15.8 (3.1)	444	ICC = 0.70	0.02
	Used any form of tobacco when first used alcohol^b^, %	68.5	62.9	143	κ = 0.64	0.47, 0.80
	Used cigarettes when first tried alcohol^b^, %	73.0	69.7	337	κ = 0.63	0.54, 0.72
	How often drink, % at least several times per week	27.2	28.1	442	ICC = 0.80	0.02
	Number of drinks when use alcohol, mean (SD)^c^	3.10 (2.3)	3.08 (2.3)	407	ICC = 0.76	0.02
	Use tobacco now when drink alcohol, % of time	59.6	58.6	917	ICC = 0.78	0.01
**Religiosity (scale 1–5)**^d^						
	My faith involves all of my life, mean (SD)	2.67 (1.5)	2.70 (1.5)	454	ICC = 0.72	0.02
	One should seek God’s guidance when making every important decision, mean (SD)	3.05 (1.6)	3.05 (1.6)	1035	ICC = 0.84	0.01
	In my life I experience the presence of the divine, mean (SD)	2.81 (1.5)	2.83 (1.5)	434	ICC = 0.70	0.02
	My faith sometimes restricts my actions.	2.39 (1.5)	2.47 (1.5)	462	ICC = 0.63	0.03
	Nothing is as important to me as serving God as best I know how, mean (SD)	2.64 (1.5)	2.68 (1.5)	452	ICC = 0.82	0.02
	I try hard to carry my religion over into all my other dealings in life, mean (SD)	2.62 (1.5)	2.66 (1.4)	463	ICC = 0.70	0.02
	My religious beliefs are what really lie behind my whole approach to life, mean (SD)	2.57 (1.5)	2.60 (1.4)	455	ICC = 0.75	0.02
	It doesn’t matter so much what I believe as long as I lead a moral life, mean (SD)	3.57 (1.5)	3.53 (1.5)	462	ICC = 0.47	0.04
	Do you participate or believe in one specific religion or belief system?, % yes	52.8	52.5	1224	κ = 0.71	0.67, 0.74
	How frequently do you attend church meetings or gatherings associated with this religion or belief system?, % daily or weekly	18.1	17.6	1224	κ = 0.55	0.51, 0.58
	How often do you pray or meditate in an effort to communicate with deity, or with what some people call a “higher power”?, % daily or weekly	49.5	50.7	1224	κ = 0.52	0.48, 0.55
	Have you ever participated in a religious or social group that discourages or prohibits tobacco use?, % yes	12.9	12.1	703	κ = 0.53	0.45, 0.60

^a^ Some questions were added to the LTUQ between first and second waves of test-retest administration, resulting in lower cell sizes.

^b^ Findings include responses of “yes,” “no,” “unsure,” or “decline to state.”

^c^ ICC calculations excluded two outliers.

^d^ Adapted from Hoge and colleagues [22,23]. Scale: 1 (“disagree”) to 5 (“agree”) plus “unsure” or “decline to state,” unless indicated otherwise in parentheses.

#### Sex, Age, and Income

Reliability estimates of several questions about the frequency of tobacco use and the age of first use differed between men and women. Statistically significant results were as follows: Reliability of self-reported age at first use was higher for women (0.84) than for men (0.78; *P* < .001). However, men’s self-reported age at onset of weekly smoking had higher reliability (0.92) than that of women (0.79; *P* < .001). Women reported pack-years with higher reliability (0.81) than did men (0.66; *P* < .001). Women also recalled the level of first-use head rush/buzz (0.60 vs 0.45 for men; *P* < .001) and difficulty inhaling (0.47 vs 0.35 for men; *P* = .02) with higher reliability (see Multimedia Appendix 3, Supplementary Table 1).

The reliability estimates of several questions varied by age group. Statistically significant results were as follows: Younger respondents (18 to 37 years) reported the age at first tobacco use less reliably (ICC = 0.77) than their older counterparts (middle, 38 to 50 years, ICC = 0.81; and older, 51 to 78 years, ICC = 0.85; *P* = .01). However, the younger group’s reporting of the age at first daily use (ICC = 0.91) showed higher reliability than that of middle (ICC = 0.82) and older (ICC = 0.79) respondents (*P* < .001). Reliability was high among all age groups for both questions. Younger respondents’ reporting of pack-years was high (ICC = 0.89), whereas the middle (ICC = 0.66) and older (ICC = 0.68) groups’ response reliability was moderate (*P* < .001). For two subjective responses to first tobacco use (irritated throat [*P* = .01], felt good [*P* = .04]), the younger group’s responses had modest and moderate reliability, while those in older groups either had modest reliability or did not meet criteria for modest reliability (see Multimedia Appendix 3, Supplementary Table 2).

The only statistically significant tobacco-use difference based on median household income was the amount of tobacco used the first time, which was reported somewhat more reliably by middle-income respondents (*P* < .001), although the reliability for all three income groups was modest (see Multimedia Appendix 3, Supplementary Table 3).

## Discussion

### Research Goals

These findings paralleled our 2-year CASI reliability study of a similar Web-based sample [13]. The present findings supported the supposition that key questions retrospectively asking about tobacco use and elements of risk and protection can be recalled with moderate to high reliability in a Web-browser environment. Potentially salient events and aspects of risk can be recalled more reliably than less memorable events (eg, age of first use of tobacco elicited higher reliability than type of tobacco first tried or amount used at first try). Sex, age, and approximated income effects in both studies indicated few reliability variations based on those characteristics.

Reliability estimates in the present 2-month study were generally higher than those reported in the 2-year test-retest reliability study of an earlier version of the LTUQ [13]. In the earlier CASI study, also conducted on a closed, invitation-only, randomly selected Web-panel convenience sample, the apparent salience of events affected reliability to a greater extent than in the present study. A 2-month test-retest administration may be more subject to carryover effects from persistence of memory, although the risk is smaller than for the shorter time intervals common in psychometric analyses of substance use questions. Some questions with low reliability in the 2-year study were not included in this study because of their apparent psychometric inadequacy.

The reliability of questions about subjective response to first use of tobacco was more modest in the 2-year study, although the present findings of scaled responses showed only modest to moderate reliability. When responses were dichotomized to *any* versus *none* in the 2-year reliability study, reliability was higher. The modest reliability of these measures suggests the advisability of neither expecting nor requiring fine-tuned recall of early events.

The relative strength of the pack-years reliability measure was comparable to that reported by Bernaards and colleagues [28], indicating that reliability of retrospective recall of pack-years can approximate that of prospective measurement, with some limitations.

The findings also provided support for exploring and expanding the use of the Web for questionnaire self-administrations. The rapid expansion of Internet access across the US population has made panel participation feasible for an increasingly broader range of respondents. A lingering question, however, is whether Internet penetration remains so linked to income and education levels that ascertaining a sufficiently broad or representative Web sample is possible. Recent findings from the Pew Internet & American Life Project [17] indicate that Internet access is no longer the domain of the young, but now crosses all age boundaries. Some 87% of those aged 30-34 use the Internet, with 83% of those aged 40-44, 80% of those aged 35-39, 80% of those 45-49, and 78% of those aged 50-54. Internet use is growing most rapidly in the 70-75 age group, with 45% currently online. Between 70% and 80% of all those online have home broadband access.

Internet access also is no longer the domain of only the wealthy and educated. As early as 2004, a commissioned research study [29] found that computer use was more than 72% for those with a high school diploma, and exceeded 86% for all other education level groups. Education attainment information collected annually through the US Census Bureau’s American Community Survey and the Current Population Survey [30] indicated that 84% of US adults older than age 25 had at least a high school diploma or equivalent. Some 54.4% had at least some college. Education attainment figures reflect some racial and ethnic disparities, such as higher education levels among Asians and lower education levels among individuals not born in the United States.

Also of concern is whether a Web sample can be representative of US smokers. A 2007 Centers for Disease Control and Prevention (CDC) report [31] indicated that smoking prevalence varied by education, with higher smoking rates among those with less than 12 years of schooling (33.3% of smokers) and those with a diploma equivalent (44.0%). Smoking rates were lower among those with more education. They were also lower among those 65 years or older (8.3%), compared with smoking rates between 21.0% and 22.8% for younger groups. Smoking rates were higher (28.8%) among those below the federal poverty level than among those above that level (20.3%). The most current census report [30] does not delineate nativity, which influences education level and could affect smoking rates.

### Limitations

#### Sample

The findings were not intended for extrapolation to the general US population or US tobacco users. Since this study was conducted on a convenience sample, its representativeness and generalizability relative to the US population were undetermined. A quota-cell, weighted, or other population-based study was beyond the scope of this research. Education level of respondents was higher than that of the US population. Race did not approximate national statistics. Additionally, the sample may have underrepresented groups still lagging in education and in Internet access, such as individuals with disabilities, those born outside the United States, and those below the federal poverty level.

The median-income approximation should be interpreted with caution because the measure, based on ZIP codes, was an indirect determination.

#### Validity

Although reliability does indicate repeatability and stability of responses, acceptable levels of reliability do not establish the validity of responses. It is possible that subjects responded consistently but inaccurately. The investigators currently are examining LTUQ validity in two longitudinal samples.

#### Pack-Years Calculation

The process of estimating pack-years from LTUQ data may have underestimated or overestimated actual total consumption. It also did not take into account the use of other types of tobacco, which would have been feasible but would have required a considerably more complex calculation. The validity of our estimation approach depended on the assumption of a linear change in the number of cigarettes smoked between any two time points for which cigarette consumption was stated. This assumption may have been particularly questionable when the individual had never succeeded in quitting for 3 or more months, and when many years separated the questionnaire administration date and the date when the individual first smoked weekly. If the ramp-up were more rapid than linear, we would have tended to underestimate pack-years. Also, if the individual temporarily reduced cigarette consumption prior to starting a quit attempt, our estimation approach would have tended to underestimate cigarette consumption for the interval ending on the date that quit attempt started. Finally, missing information about other quit attempts (of any duration) may have resulted in overestimation of pack-years.

### Conclusions

This study reinforced the expectation that retrospectively collected self-report data about the development and maintenance of addictive behaviors can be a valuable and reliable source of information about lifetime substance use [4]. The present results add to the evidence indicating that this relatively economical approach can yield reliable reports of behaviors that have not been captured in real time. The findings thus provide support for exploring and expanding the use of the Web for questionnaire self-administrations.

As Internet penetration breaks through demographic boundaries, sampling can more readily include those with less education, lower income levels, and those in older age ranges. Even so, in spite of greater relative ease of access, accurate Web-based research will continue to require appropriate sampling and analytic procedures, as well as cautious interpretation and extrapolation.

## Multimedia Appendix 1

Screen views from the LTUQ

## Multimedia Appendix 2

Evaluating data integrity in retrospective recall of lifetime tobacco use

## Multimedia Appendix 3

Supplementary tables

## Abbreviations

CASI: computer-assisted self-interviewing
LTUQ: Lifetime Tobacco Use Questionnaire
