# Supplementary material for: Test-Retest Reliability of Web-Based Retrospective Self-Report of Tobacco Exposure and Risk
Source: J Med Internet Res. 2009 Aug 11;11(3):e35. doi: 10.2196/jmir.1248 (PMC2762856; doi:10.2196/jmir.1248)
Supplement: Supplementary file 1 [file jmir_v11i3e35_app1.pdf]

Samples of LTUQ Screens

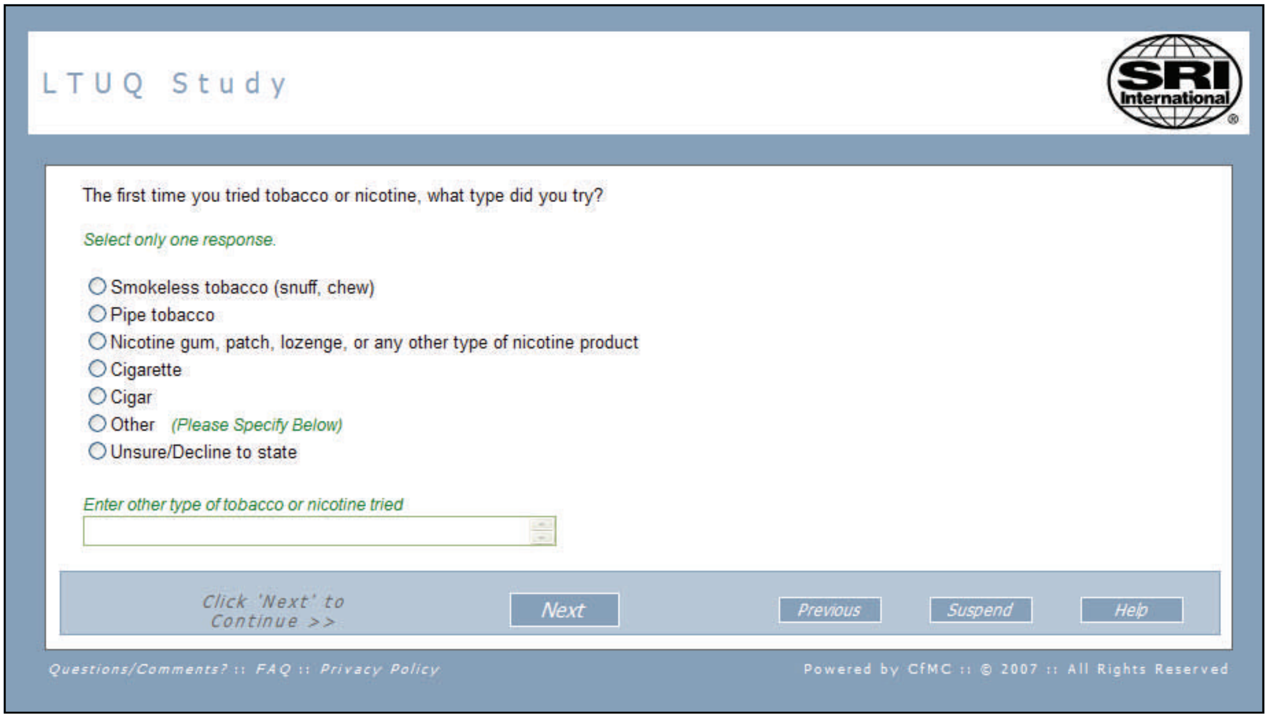

Figure 1. LTUQ question about first use of tobacco. The LTUQ includes specific questions about four major types of tobacco (cigarettes, cigars, pipe, and smokeless), and allows open-ended responses regarding other types of tobacco and nicotine. Response option order was randomized across administrations, with the two final options anchored.

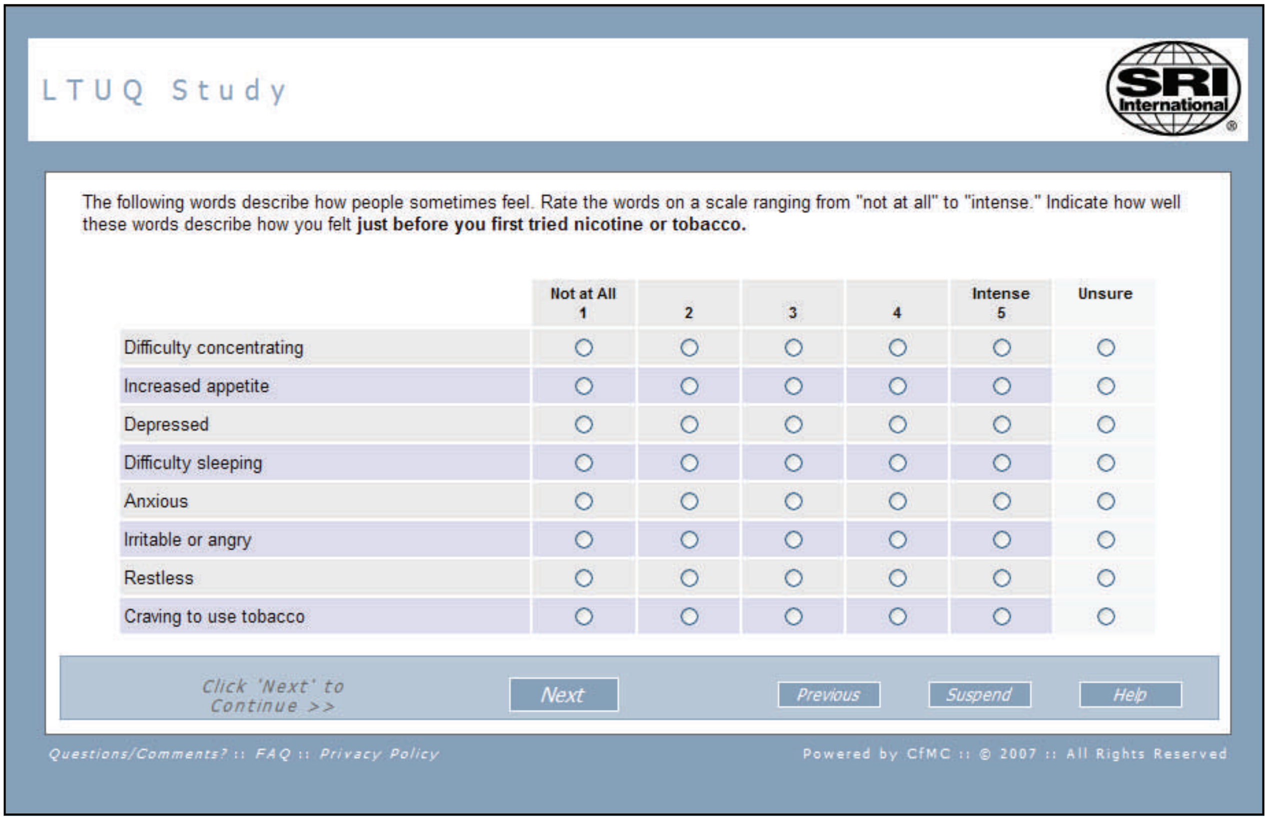

Figure 2. LTUQ grid questions about abstinence symptoms at baseline, before first tobacco use. Row order was randomized across administrations to avoid order effects.

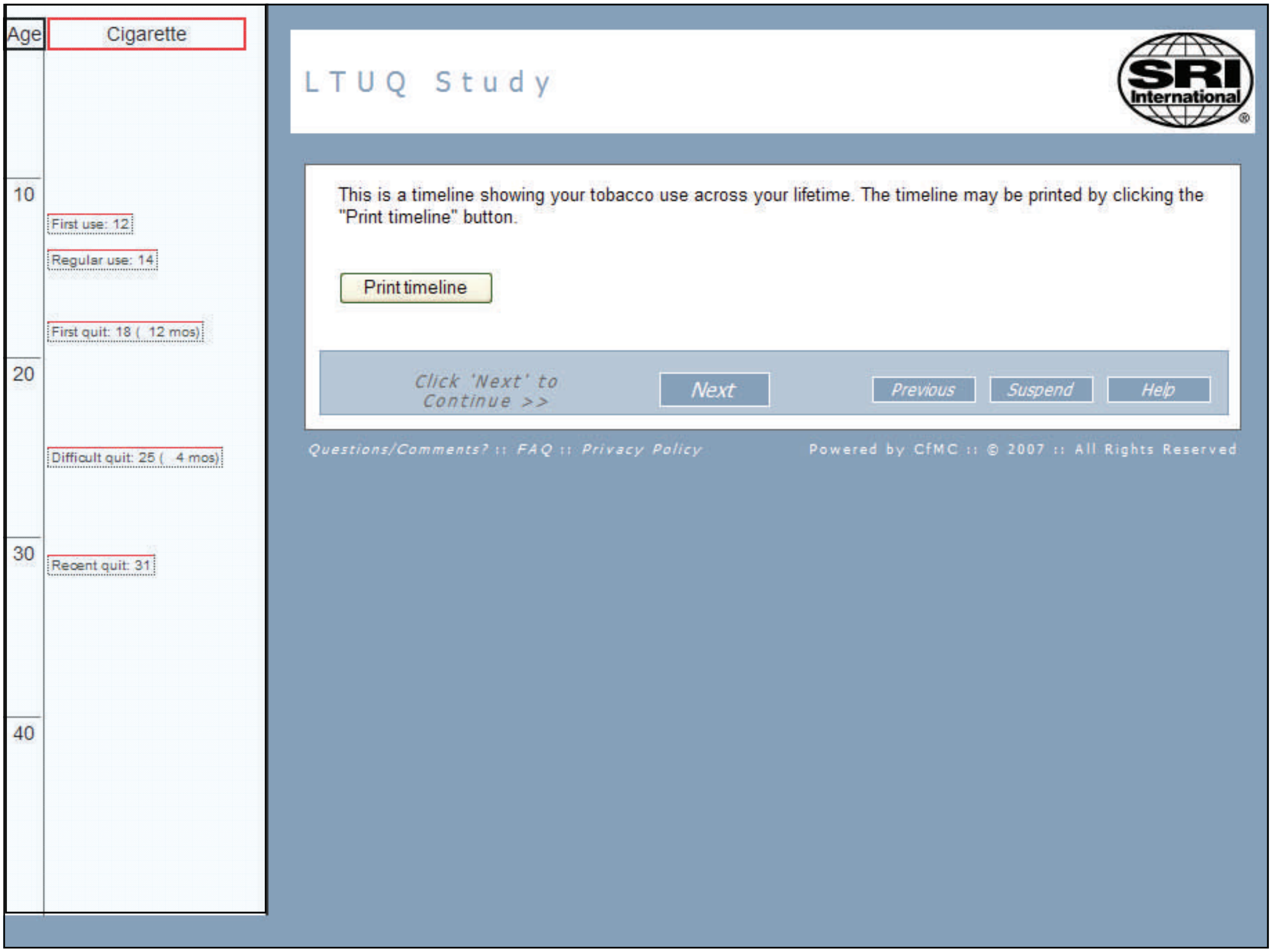

Figure 3. An interactive timeline was generated throughout the administration of the LTUQ, to provide visual depiction and verification of information provided by a respondent.

This screen shows that the only form of tobacco used was cigarettes, that the first use of cigarettes was at age 12, that regular use (defined here as weekly) started at age 14, that the first quit attempt was at age 18 and lasted 12 months, that the respondent's most difficult quit attempt occurred at age 25 and lasted 4 months, and that the most recent quit attempt was at age 31. (Quit attempt difficulty was an experimental variable not reported in the present study.)

The respondent could use the Previous button to change earlier responses, could suspend the questionnaire and resume with a password, could request help, or could continue. The timeline was printable.
