# Supplementary material for: Test-Retest Reliability of Web-Based Retrospective Self-Report of Tobacco Exposure and Risk
Source: J Med Internet Res. 2009 Aug 11;11(3):e35. doi: 10.2196/jmir.1248 (PMC2762856; doi:10.2196/jmir.1248)
Supplement: Supplementary file 2 [file jmir_v11i3e35_app2.pdf]

Evaluating Data Integrity in Retrospective Recall of Lifetime Tobacco Use

Janet Brigham, Christina N. Lessov-Schlaggar, Ruth Krasnow, Harold S. Javitz, and Gary E. Swan
Center for Health Sciences, SRI International, Menlo Park, California USA

BACKGROUND

The expansion and penetration of Web access throughout much of the world makes Web-based research tools an affordable option. Additionally, a developing body of research indicates that respondents may be more open about reporting less socially acceptable behaviors when interacting with a device rather than an interviewer.

However, as Web-based research strategies draw on anonymous respondents, particularly through the less optimal opt-in approach rather than invitation-only panels, the risk of compromised data integrity increases. This underscores the need to monitor data for response patterns associated with inadequate responses. This often is identified by inconsistency, too-rapid responses, and straight-line answers on grid questions.

METHODS

We examined ways to monitor and identify problems with data integrity in administering the Lifetime Tobacco Use Questionnaire (LTUQ), a Web-based, password-controlled questionnaire that assesses tobacco use retrospectively across the lifespan. The LTUQ examines the use of nicotine and tobacco from earliest exposure and initiation through current use, with retrospective and current-use questions. The LTUQ probes recall of the use of four primary types of tobacco (cigarettes, cigars, smokeless, and pipe) and has a limited question set about additional types of tobacco. It provides an interactive timeline that allows the respondent to verify timeline information he or she has provided:

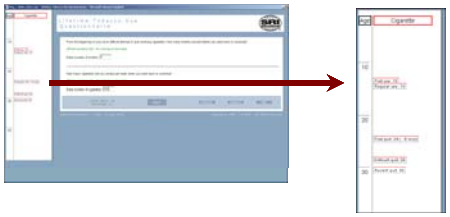

The LTUQ is programmed with validity and consistency checks that can be turned off or on depending on research goals. For most applications of the LTUQ, the consistency checks are turned on for basic questions such as age, age at first use of tobacco, and age at first regular use (as defined by investigator-determined values). For the present studies, those consistency checks were not engaged, so that we could evaluate the consistency of responses as part of the overall identification of data integrity. Grid questions in this report involved rating subjective responses to first use of tobacco and to withdrawal symptoms.

Studies

We studied the psychometric properties of core LTUQ questions and modules, including responses to first use of tobacco, in two studies:

- A two-month test-retest reliability study of 494 tobacco users in January and March 2006.
- A sample of 2,406 tobacco users (at least 100 cigarettes lifetime) in August 2006. Of the expected 26% of smokers among the 6,452 invited, 37% completed the LTUQ.

Respondents to both studies were recruited from an invitation-only (not opt-in) Web panel drawn from consumer databases.

STUDY 1: Test-retest examination of retrospective reporting reliability (*N* = 494)

- ***Recruitment:*** A sample of male and female Web panel members was randomly invited to complete the LTUQ.
- ***Respondents:*** Adult (19 to 78 years old; mean, 43.2 years) 46.4% female, 87.2% White, 69.6% some college, associate or bachelor degree, limited here to those using 100 cigarettes or more lifetime.
- ***Procedure:*** Respondents completed the LTUQ at Time 1 in January 2006, then were invited to complete it a second time in March 2006. Invitations were sent by e-mail from the panel sample provider; at both completions, respondents were provided points redeemable for a monetary incentive.
- ***Research questions:***
  1. Which cases involved different respondents at Times 1 and 2 (contrary to instructions given to respondents)?
  2. Which respondents at either or both Times 1 and 2 paid inadequate attention to the questionnaire items?

STUDY 2: Sample of smokers reporting retrospectively about lifetime tobacco use (*N* = 2,406)

- ***Recruitment:*** Previously unrecruited members of the Web panel were randomly selected and invited to complete a screening question eliminating those who had not used 100 cigarettes lifetime.
- ***Respondents:*** Adult (18 to 80 years old; mean, 43.6 years) 56.3% female; 88.1% White; 72.4% some college, associate, or bachelor degree; respondents had used at least 100 cigarettes lifetime.
- ***Procedure:*** Respondents completed the LTUQ in August 2006. Participants were provided points redeemable for a monetary incentive.
- ***Research question:*** Which respondents did not attend adequately to the questionnaire, evidenced by rapid response to grid questions and straight-line responding?

RESULTS

STUDY 1: Test-retest (*N* = 494)

Responses at both testing times were compared on the basis of age and gender. A total of 17 (3.4%) reported current age differing by more than 2 years (allowing for respondent rounding in age reporting) across the retest interval. Some 16 (3.2%) of respondents reported different gender at Time 1 vs. Time 2. We also examined median overall completion time, comparing (a) participants whose age and gender matched in both testing times (*n*=478) vs. (b) cases (*n*=22) whose gender did not match and whose age differed by more than 2 years at the two testing times.

Median completion time for those with mismatched Time 1-Time 2 age or gender responses (invalid cases, *n* = 22) was 10.2 min at Time 1 and 10.5 min at Time 2. In comparison, median completion time for valid cases was 16.1 min at Time 1 and 14.7 min at Time 2.

STUDY 2: Single sample of smokers (*N* = 2,406)

We examined mean completion times for grid questions and examined straight-line reporting (in which respondents answered all questions in a grid with the same response, not *unsure/decline*).

- 17 respondents answered at least two of three timed grids with less than 1 second per response. Of these 17 respondents, 13 provided straight-line answers (e.g., all 2's, all 3's; not *unsure/decline*) on at least two timed grids.
- Median total questionnaire completion time for the 15 straight-line, most rapid responders was 5.22 min; all other respondents' median total completion time was 14.0 min.

CONCLUSIONS

- Potentially invalid responses can be identified in test-retest studies by comparing independent variables (such as key demographics) across time. Whether these represent mismatched respondents or inadequate attention, excluding invalid responses can help maintain research integrity.
- Computerized testing that reports response times on grid questions or other question groups can identify respondents whose overall patterns reflect potentially inadequate responses. However, it is advisable to use more than one measure of quality, such as timing *and* response pattern.

Correspondence:
Janet Brigham, Ph.D., Center for Health Sciences, SRI International,
333 Ravenswood Ave., Menlo Park, CA, 94025 (janet.brigham@sri.com).
This research is sponsored by NIDA (DA11170). Sample provided by eRewards; programming and Web hosting by CfMC Research Software.
