# Supplementary material for: Test-Retest Reliability of Web-Based Retrospective Self-Report of Tobacco Exposure and Risk
Source: J Med Internet Res. 2009 Aug 11;11(3):e35. doi: 10.2196/jmir.1248 (PMC2762856; doi:10.2196/jmir.1248)
Supplement: Supplementary file 3 [file jmir_v11i3e35_app3.pdf]

Supplementary Table 1. Sex-based comparison of 2-month test-retest reliability of self-report of respondent characteristics and tobacco use.

	Men (n = 590)		Women (n = 634)		Sex comparison			
	**ICC or κ**	**(CI) or SE**	**ICC or κ**	**(CI) or SE**	**Reliability difference**	**CI for difference**	**t test**	***P* value**
**Respondent characteristics**								
Education	κ = 0.89	(0.86, 0.92)	κ = 0.90	(0.88, 0.93)	0.017	-0.02, 0.06	0.69	.41
Race/ethnicity[a]	κ = 0.86	(0.80, 0.91)	κ = 0.88	(0.82, 0.94)	0.016	-0.07, 0.10	0.15	.70
**Frequency of tobacco use**								
Cigarettes	κ = 0.49	(0.38, 0.59)	κ = 0.34	(0.21, 0.46)	0.150	-0.31, 0.01	3.33	.07
Cigars	κ = 0.47	(0.36, 0.58)	κ = 0.38	(0.20, 0.55)	0.095	-0.30, 0.11	0.80	.37
Smokeless	κ = 0.61	(0.49, 0.74)	κ = 0.56	(0.34, 0.78)	0.050	-0.31, 0.21	0.15	.70
Pipe	κ = 0.49	(0.35, 0.63)	κ = 0.55	(0.31, 0.79)	0.061	-0.22, 0.34	0.19	.67
**Early use of tobacco**								
Age first tobacco use	ICC = 0.78	0.02	ICC = 0.84	0.01	0.060	0.02, 0.10	9.00	.00
Type of tobacco first tried	κ = 0.51	(0.37, 0.65)	κ = 0.28	(-0.16, 0.72)	0.227	-0.69, 0.23	0.93	.34

(Supplementary Table 1, continued)

	Male (n = 590)		Female (n = 634)		Sex comparison			
	ICC	SE	ICC	SE	Reliability difference	CI for difference	*t* test	*P* value
**Experienced at first use of tobacco, scaled score (1 – 5)**								
Dizzy	0.46	0.03	0.55	0.03	0.09	-0.01, 0.18	3.41	.07
Lightheaded	0.46	0.03	0.52	0.03	0.06	-0.03, 0.16	1.82	.18
Nauseated	0.56	0.03	0.53	0.03	0.03	-0.12, 0.05	0.58	.45
Enjoyed it	0.50	0.03	0.51	0.03	0.02	-0.07, 0.11	0.15	.70
Coughing/choking	0.52	0.03	0.50	0.03	0.03	-0.12, 0.06	0.32	.57
Liked taste	0.55	0.03	0.47	0.04	0.08	-0.17, 0.01	2.99	.08
Felt bad	0.51	0.03	0.50	0.03	0.01	-0.11, 0.08	0.08	.78
Relaxed/calm	0.37	0.04	0.34	0.04	0.03	-0.14, 0.08	0.26	.61
Irritated throat	0.52	0.03	0.46	0.04	0.06	-0.15, 0.04	1.40	.24
Head rush/buzz	0.45	0.04	0.60	0.03	0.15	0.06, 0.24	11.3	.00
Felt good	0.40	0.04	0.35	0.04	0.05	-0.16, 0.06	0.83	.36
Difficulty inhaling	0.35	0.04	0.47	0.04	0.12	0.02, 0.22	5.17	.02
Liked smell	0.54	0.03	0.45	0.04	0.09	-0.18, 0.01	3.43	.06
**Weekly use of cigarettes**								
Age first smoked at least weekly	0.92	0.01	0.79	0.02	0.14	-.18, .10	46.5	.00
Cigarettes/week at first weekly smoking (male, *n* = 232; female, *n* = 322)	0.49	0.04	0.52	0.05	0.04	-.09, .16	0.30	.59
**Daily use of cigarettes**								
Age at first used daily, years	0.81	0.02	0.84	0.02	0.04	-.01, .08	2.55	.11
Cigarettes/day when started daily use (male, *n* = 210, female, *n* = 305)	0.42	0.05	0.46	0.05	0.04	-.10, .18	0.38	.54
**Pack-years**	0.66	0.03	0.81	0.02	0.15	.07, .23	13.69	.00

Note. *CI*, confidence interval; *ICC*, intraclass correlation coefficient; *SE*, standard error; [a]Includes responses of *unsure* or *decline to state*. Reliability was not calculated for respondent characteristic variables Age and Sex because variables were used for screening.

Supplementary Table 2. Age group comparison of 2-month test-retest reliability of self-report of respondent characteristics and tobacco use.

	Younger (n = 422)		Middle (n = 400)		Older (n = 402)		Age comparison	
	**ICC or κ**	**(CI) or SE**	**ICC or κ**	**(CI) or SE**	**ICC or κ**	**(CI) or SE**	**Chi-square**	***P value***
**Respondent characteristics**								
Education	κ = 0.88	(0.84, 0.91)	κ = 0.91	(0.88, 0.94)	κ = 0.91	(0.87, 0.94)	2.24	.33
Race/ethnicity	κ = 0.90	(0.86, 0.95)	κ = 0.91	(0.84, 0.99)	κ = 0.89	(0.79, 0.99)	0.09	.96
**Frequency of tobacco use**								
Cigarettes	κ = 0.39	(0.26, 0.51)	κ = 0.47	(0.33, 0.61)	κ = 0.40	(0.24, 0.57)	0.83	.66
Cigars	κ = 0.46	(0.30, 0.62)	κ = 0.33	(0.15, 0.51)	κ = 0.53	(0.39, 0.68)	2.86	.24
Smokeless	κ = 0.56	(0.41, 0.72)	κ = 0.52	(0.31, 0.74)	κ = 0.81	(0.63, 0.99)	5.55	.06
Pipe	κ = 0.49	(0.29, 0.70)	κ = 0.38	(0.11, 0.66)	κ = 0.58	(0.41, 0.76)	1.51	.47
**Early use of tobacco**								
Age at first tobacco use	ICC = 0.77	0.02	ICC = 0.81	0.02	ICC = 0.85	0.01	9.61	.01
Type of tobacco first tried	κ = 0.39	(0.22, 0.55)	κ = 0.30	(0.12, 0.48)	κ = 0.30	(0.10, 0.51)	0.65	.72
**Weekly use of cigarettes**								
Age first smoked at least weekly	ICC = 0.90	.01	ICC = 0.90	0.01	ICC = 0.89	0.01	0.34	.85
Cigarettes/week at first weekly smoking (younger = 152, middle = 180, older= 222)	ICC = 0.37	.07	ICC = 0.49	0.06	ICC = 0.56	0.05	5.32	.07

(Supplementary Table 2, continued)

	Younger (n = 422)		Middle (n = 400)		Older (n = 402)		Age comparison	
	ICC or κ	(CI) or SE	ICC or κ	(CI) or SE	ICC or κ	(CI) or SE	Chi-square	*P value*
**Daily use of cigarettes**								
Age first used daily, years	ICC = 0.91	0.01	ICC = 0.82	0.02	ICC = 0.79	0.02	33.73	.00
Cigarettes/day when started daily use (younger = 133, middle = 169, older = 213)	ICC = 0.43	0.07	ICC = 0.39	0.07	ICC = 0.52	0.05	2.65	.27
Pack-years	ICC = 0.89	0.02	ICC = 0.66	0.04	ICC = 0.68	0.04	39.3	.00
**Experienced at first use of tobacco (scaled score 1–5)**								
Dizzy	ICC = 0.48	0.04	ICC = 0.47	0.04	ICC = 0.56	0.04	3.00	.22
Lightheaded	ICC = 0.48	0.04	ICC = 0.43	0.04	ICC = 0.55	0.04	4.37	.11
Nauseated	ICC = 0.55	0.04	ICC = 0.53	0.04	ICC = 0.55	0.04	0.16	.92
Enjoyed it	ICC = 0.56	0.04	ICC = 0.45	0.04	ICC = 0.50	0.04	3.61	.16
Coughing/choking	ICC = 0.56	0.04	ICC = 0.51	0.04	ICC = 0.47	0.04	2.44	.30
Liked taste	ICC = 0.55	0.04	ICC = 0.50	0.04	ICC = 0.47	0.04	1.81	.41
Felt bad	ICC = 0.49	0.04	ICC = 0.52	0.04	ICC = 0.50	0.04	0.24	.89
Relaxed/calm	ICC = 0.42	0.05	ICC = 0.35	0.05	ICC = 0.28	0.05	3.84	.15
Irritated throat	ICC = 0.59	0.04	ICC = 0.45	0.04	ICC = 0.44	0.04	9.39	.01
Head rush/buzz	ICC = 0.51	0.04	ICC = 0.53	0.04	ICC = 0.47	0.04	1.14	.57
Felt good	ICC = 0.46	0.04	ICC = 0.28	0.05	ICC = 0.39	0.05	6.31	.04
Difficulty inhaling	ICC = 0.46	0.04	ICC = 0.36	0.05	ICC = 0.41	0.05	2.29	.32
Liked smell	ICC = 0.50	0.04	ICC = 0.52	0.04	ICC = 0.47	0.04	0.79	.68

Note. CI, confidence interval; ICC, intraclass correlation coefficient; SE, standard error; κ = kappa. Reliability was not calculated for respondent characteristic variables Age and Sex because variables were used for data integrity screening.

Supplementary Table 3. Median household hold income approximation comparison of test-retest reliability of self-report of respondent characteristics and tobacco use.

	Lower (n = 398) M = $34,530, SD = $5,160		Middle (n = 398) M = $48,678, SD = $4,466		Higher (n = 396) M = $70,895, SD = $13,200		Median income comparison	
	**Reliability estimate**	**CI**	**Reliability estimate**	**CI**	**Reliability estimate**	**CI**	**Chi-square**	***P* value**
**Respondent characteristics**								
Age	*r* = 0.99	.99, .99	*r* = 0.99	0.99, 0.99	*r* = 0.99	0.99, 0.99		
Education	κ = 0.91	.87, .94	κ = 0.89	0.86, 0.93	κ = 0.89	0.85, 0.92	0.57	.75
Race/ethnicity	κ = 0.86	.78, .94	κ = 0.90	0.84, 0.97	κ = 0.98	0.94, 1.00	9.53	<.01
**Frequency of tobacco use**								
Cigarettes	κ = 0.43	.29 .58	κ = 0.42	0.27, 0.58	κ = 0.42	0.29, 0.55	0.01	.99
Cigars	κ = 0.54	.36, .72	κ = 0.26	0.09, 0.43	κ = 0.46	0.31, 0.61	5.40	.07
Smokeless	κ = 0.76	.60, .91	κ = 0.46	0.21, 0.71	κ = 0.57	0.39, 0.75	4.87	.09
Pipe	κ = 0.70	.51, .88	κ = 0.35	0.11, 0.58	κ = 0.43	0.23, 0.63	10.1	<.01
**Early use of tobacco**								
Age first tobacco use	*r* = 0.87	.84, .89	*r* = 0.82	0.78, 0.85	*r* = .88	0.85, 0.90	2.08	.35
Type of tobacco first tried, % cigarette	κ = 0.63	.39, .86	κ = 0.42	0.18, 0.66	κ = 0.49	0.27, 0.72	1.42	.49
Amount used at first try[a]	κ = 0.36	0.25, 0.47	κ = 0.49	0.37, 0.61	κ = 0.28	0.15, 0.40	21.6	<.001

Note. CI, confidence interval, M, mean, SD, standard deviation. [a]Includes responses of *unsure* or *decline to state*. Otherwise excludes responses of *unsure* or *decline to state*.
